# Non-surgical treatment of dentin caries in preschool children – systematic review

**DOI:** 10.1186/s12903-015-0033-7

**Published:** 2015-04-03

**Authors:** Duangporn Duangthip, Ming Jiang, Chun Hung Chu, Edward CM Lo

**Affiliations:** Faculty of Dentistry, University of Hong Kong, 3/F, Prince Philip Dental Hospital, 34 Hospital Road, Hong Kong, SAR China

**Keywords:** Dental caries, Primary teeth, Preschool children, Fluoride, Treatment, Review

## Abstract

**Background:**

Untreated dentin caries in primary teeth is commonly found in preschool children worldwide. Recently, the use of simple non-surgical approaches to manage the situation has been advocated.

The aim of the study was to systematically review and evaluate the literature on effectiveness of non-surgical methods in arresting or slowing down the progression of active dentin caries in primary teeth in preschool children.

**Methods:**

A systematic search of the main electronic databases (Pubmed, Cochrane Collaboration, EMBASE) was conducted to identify peer reviewed papers published in English in the years 1947–2014. Keywords and MeSH terms used in the search were “dental caries”, “primary dentition” and various non-surgical treatments (fluoride, sealant, resin infiltration, xylitol, chlorhexidine, CPP-ACP, ozone, etc.). The inclusion criteria were clinical studies conducted in children under 6 years old, and reported findings on caries arrest or caries progression in primary teeth. Retrieved papers were read by two reviewers independently to assess suitability for inclusion, and the final decision was made by consensus. Quality of the included studies was assessed and data were extracted for analysis.

**Results:**

The search identified 323 papers for screening. Among these, 290 papers did not satisfy the study inclusion criteria. Consequently, 33 full papers were retrieved and reviewed. Finally, 4 studies were included. Three studies reported that topical applications of silver diammine fluoride (SDF) solution could arrest dentin caries in preschool children. One study supported that having a daily toothbrushing exercise in kindergarten using toothpaste with 1000 ppm fluoride could stabilize the caries situation in young children.

**Conclusions:**

There is limited evidence to support the effectiveness of SDF applications or daily toothbrushing with fluoride toothpaste in arresting or slowing down the progression of active dentin caries in primary teeth in preschool children. More well-designed randomized controlled trials are required to confirm these findings.

## Background

Dental caries remains one of the most common childhood diseases worldwide. Beneficial effects of dental rehabilitation on dental and general health of children with dental caries have been reported [1]. Dental fillings or restorations have been used as a treatment option for the management of early childhood caries (ECC) [2]. However, this procedure requires sophisticated equipment and well trained dental personnel, especially when treating apprehensive young children. Studies have found ECC being more prevalent among children coming from lower socio-economic groups and high proportions of the cavitated carious primary teeth in preschool children are left untreated [3]. The dental profession is currently faced with an enormous task of how to manage the huge burden of consequences of the caries process amongst the world population [4]. Dentists have barriers to the treatment of ECC [5]. Providing care for young children can be stressful and troublesome. Despite clear guidelines that restorations should be provided, the feasibility of asking general dental practitioners to restore all the decayed primary teeth has been questioned [6]. Fortunately, the requirements for management of caries in the primary dentition can be different from those in the permanent dentition as the lifespan of primary teeth before tooth shedding is usually about 6–8 years [7].

Currently, there is growing evidence that minimally invasive approaches can arrest caries progression such that the involved primary tooth can remain in the mouth till exfoliation without causing the child pain and infection. Caries arrest treatment with fluorides in various vehicles (toothpaste, gel, varnish, soluation, mouthrinse) has been shown to be a viable alternative to the traditional restorative approach. Studies showed that daily toothbrushing using fluoridated toothpaste (1000 ppm F) could arrest non-cavitated lesions [8] as well as dentin caries lesions [9]. Toothpaste containing higher fluoride concentration, e.g. 5000 ppm, has better results in remineralizing carious lesions compared to those containing 1000 ppm [10]. A number of studies in children have shown that silver diammine fluoride (SDF) solution is effective in preventing dental caries [11,12] and in arresting dental caries [13-15]. Although SDF has been used for more than four decades with no reported complications, adoption by clinicians is still limited, probably due to the black staining associated with the arrested caries lesions [16].

Different modalities for treating caries lesions have been proposed. These include pit and fissure sealants, resin infiltration, xylitol, chlorhexidine, casein phosphopeptide - amorphous calcium phosphate (CPP-ACP) and ozone therapy. The use of fissure sealant over carious lesion as a therapeutic intervention was suggested in the 1970s [17]. Despite the possibility of clinical success in caries arrest, the use of sealant as a therapeutic treatment for caries into dentin is still controversial [18]. An innovative approach to arrest progression of caries in enamel is recently introduced, in which a low-viscosity resin (infiltrant) is used to infiltrate and seal non-cavitated interproximal surface carious lesions in permanent teeth [19]. CPP-ACP is suggested as a promising remineralizing agent with a significant effect which has been demonstrated in both in vivo and in vitro studies [20] but the advantage of using CPP-ACP as a supplement to fluoride-containing products is still unclear. Xylitol is non-cariogenic and has a dose-frequency-dependent antimicrobial effect on mutans streptococci [21]. Studies on the caries-preventive effect of xylitol in children have been published [22,23]. In summary, a number of novel non-surgical treatment options are being developed and debated for the management of caries in permanent teeth. However, the clinical evidence of these techniques for primary teeth is still unclear.

Untreated dental caries is a global pandemic in young children [24]. Thus, instead of placing dental restorations, arresting dental caries might meet the requirement of new global oral health goals due to the remarkable benefits such as affordable cost and simplicity to implement [25]. However, the generalizability of using these alternative treatments in young children has been questioned since the success of a treatment for decayed primary teeth also depends on children’s behaviors. To date, there is a lack of scientific evidence for clinically-effective non-surgical caries management, focusing on primary teeth in preschool children. This systematic review aimed to assess the effectiveness of non-surgical treatments of dentin caries in primary teeth in preschool children.

## Methods

Criteria for considering studies for this review are listed below:

### Type of studies

Clinical studies of randomized controlled trials, controlled trial and longitudinal observation (prospective or retrospective study) with a minimum period of 6 months were included.

### Type of participants

Children aged 6 or below who had at least one dentin carious lesion in the primary dentition at the start of the study were considered for inclusion in this review.

### Type of interventions and outcomes

Various non-surgical intervention methods such as fluoride agents (toothpaste, mouthrinse, gel, varnish, solution), dental sealant, resin infiltrant, chlorhexidine (CHX), xylitol, CPP-APC, ozone and oral health education were included.

The primary outcomes of the included studies were caries arrest, progression or regression. There could be comparisons of outcomes of different non-surgical approaches or comparisons of outcomes of non-surgical and surgical approaches. The treatment could be performed by dentists or dental auxiliaries. The location of treatment could be in any place such as in kindergarten, hospital or dental clinic.

### Exclusion criteria for considering studies for this review

A paper was excluded if it was in one or more of the following categories:

1. incomplete description of sample selection and outcomes or poor study design;
2. early reports of studies, in-vitro or animals studies, narrative reviews or systematic reviews.

Where doubt existed over the exclusion of a study based on the title or abstract, full paper was retrieved.

### Search strategy

Identification of studies to be considered for inclusion was based on a systematic search on the common electronic databases such as Pubmed, Cochrane collaboration and EMBASE. The search was restricted to reports written in English published from 1947 to June 2014. Reports in the gray literature such as dissertations, theses, unpublished studies, product reports were not included. Inclusion and exclusion criteria were applied by examining the title and abstracts. The identified studies were independently reviewed by two reviewers for eligibility.

The keywords and MeSH terms were combined using four main concepts:

1. dental caries [MeSH Term] OR tooth demineralization [MeSH Term]
2. primary dentition [MeSH Term] OR “deciduous teeth” OR “deciduous tooth“ OR “milk teeth” OR “milk tooth” OR “primary teeth” OR “primary tooth” OR child [MeSH Term]
3. fluorides [MeSH Term] OR pit and fissure sealants [MeSH Term] OR xylitol [MeSH Term] OR chlorhexidine [MeSH Term] OR “casein phosphopeptide-amorphous calcium phosphate” [Supplementary Concept] OR ozone [MeSH Term] OR cariostatic agents [MeSH Terms] OR “resin infiltration”
4. “progress*” OR “arrest*” OR “caries arrest” OR “caries progression” OR “caries control”.

Since papers on the sole use of silver compounds, such as silver nitrate, without combination with fluorides would not be captured by the search using the MeSH term “fluorides”, an additional search using a combination of the following MeSH terms, “silver compounds” AND “dental caries” AND “primary teeth”, was carried out (last search on 29 January 2015).

### Assessment criteria

Assessment of risk of bias in the included studies was conducted by using the Cochrane risk of bias assessment tool [26]. Seven domains were assessed for each included study: sequence generation, allocation concealment, masking of participants and personnel, masking of outcome assessment, incomplete outcome data, selective outcome reporting and other bias. Within each domain, a judgement of ‘low’, ‘high’ or ‘unclear’ risk of bias was made. An overall risk of bias was also made as follows:

- low risk of bias (plausible bias unlikely to seriously alter the results)
- unclear risk of bias where one or more of the domains were assessed as unclear
- high risk of bias (plausible bias that weaken confidence in the results) where one or more domains were assessed at high risk of bias.

The studies were graded as good, fair or poor based on the ADA’s criteria after assessment of their quality using the following criteria reported in the ADA Clinical Recommendations Handbook [27]:

- initial assembly of comparable groups
- consideration of potential confounders with either restriction or measurement for adjustment in the analysis; consideration of inception cohorts
- maintenance of comparable groups (includes attrition, cross-over, adherence, contamination)
- important differential loss to follow-up or overall high loss to follow-up
- measurements: equal, reliable, and valid (includes masking of outcome assessment)
- clear definition of interventions
- all important outcomes considered
- analysis: adjustment for potential confounders for cohort studies, or intention to treat analysis for RCTs.

### Data extraction

The indentified papers were reviewed independently by two investigators who were not involved in any of the included studies. The extracted information was compared and reporting was decided by consensus. If in doubt, a senior investigator was consulted. The primary summary measure for reporting in this review was the success rates of the various treatments. Regarding the treatment effect, the number needed to treat (NNT) was the average number of active caries surfaces that needed to be treated for one to benefit, i.e. become arrested, compared with the control [28]. NNT was calculated from the original data according to the following formula:

1. $$ \mathrm{Proportion}\ \mathrm{benefiting}\ \mathrm{from}\ \mathrm{treatment}\ \left[\mathrm{P}\left(\mathrm{c}\right)\right] = \mathrm{Caries}\ \mathrm{arrest}\ \mathrm{rate}\ \mathrm{in}\ \mathrm{experiment}\ \mathrm{group}\ \left[\ \mathrm{P}\left(\mathrm{a}\right)\right]\ \hbox{--}\ \mathrm{Caries}\ \mathrm{arrest}\ \mathrm{rate}\mathrm{in}\ \mathrm{control}\ \mathrm{group}\ \left[\mathrm{P}\left(\mathrm{b}\right)\right] $$
2. $$ \mathrm{N}\mathrm{N}\mathrm{T} = 1/\ \mathrm{Proportion}\ \mathrm{benefiting}\ \mathrm{from}\ \mathrm{treatment}\ \left[\mathrm{P}\left(\mathrm{c}\right)\right] $$

## Results

**Figure 1:**
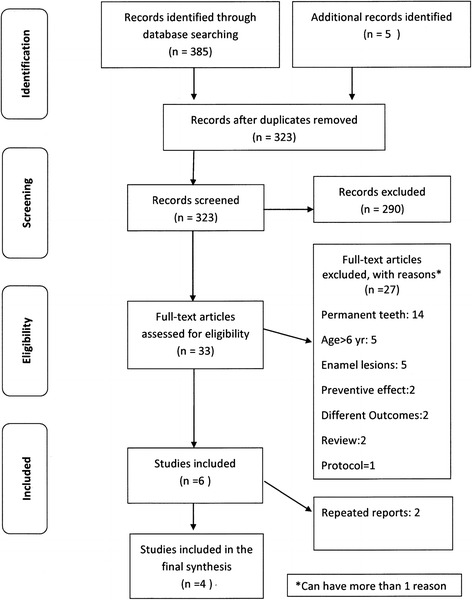
**Flow diagram of identification and study selection for qualitative synthesis.**

From the search in the PubMed, Cochrane Collaboration and EMBASE electronic databases, a total of 385 records were initially found. Five additional papers in English were identified using a combination of the MeSH terms, “Silver Compounds” AND “Dental Caries” AND “Primary teeth”. Then, 323 de-duplicated records were checked on the basis of the title, keywords, and abstract. Among these, 290 records did not meet the inclusion criteria, such as conducted on permanent teeth, report on preventive effect only, only enamel caries treated, review papers and in-vitro studies. Consequently, 33 full papers were retrieved and reviewed. Twenty-seven papers were excluded due to one or more of the following reasons: conducted on permanent teeth (51.9%), age of participants was over 6 years (18.5%), only enamel lesions treated (18.5%), reported on preventive effect only (7.4%), review paper (7.4%), different outcomes (7.4%), reported on protocol only (3.7%) and small sample size (3.7%). Among the 6 included papers, 2 papers reporting on the same study were removed [29,30]. Thus, four studies were included in the final report. Flow chart of identification and study selection for qualitative synthesis is shown in Figure 1. Details of the included studies about non-surgical approaches in treating dentin caries in preschool children are summarized in Table 1.

**Table 1:** **A summary of included studies on non-surgical approaches in treating dentin caries in preschool children**

**Author/year**	**Study type/duration**	**Subjects**	**Intervention**	**Outcome assessment**	**Results/conclusion**	**Comment/quality assessment**
Zhi et al., 2012 [15]	RCT	212 children	Gp 1: 38% SDF once a year	Clinical examination criteria:	At 1 year, the caries arrest rates of Gps 1, 2 and 3 were 37%, 53% and 29%, respectively.	Well-planned study design (random allocation, adequate sample size) Dropout rate was not high (15%). Confounding factors were taken into consideration.
	2 years	aged 3–4 years with active caries in primary teeth	Gp 2: 38% SDF twice a year	Active: lesion easily penetrated by probe		
			Gp 3: Flowable GI filling once a year	Arrested: smooth, hard surfaces when probing	At 2 years, the caries arrest rates of three groups were 79%, 91% and 82%, respectively.	
				But for Gp 3 also lesions that were totally covered with GI	Effect of annual SDF and GI application on arresting caries did not differ significantly.	However, blinding of outcome assessment (between Gp 3 and Gp 1, 2) is impossible.
					SDF application twice a year increased caries arrest rates.	*Low risk of bias*
						*Study quality (ADA): good*
dos Santos et al., 2012 [14]	RCT	91 children aged 5–6 years with caries in primary teeth	Gp 1: interim restorative treatment with GI filling without caries removal	Clinical examination criteria:	At 12 months, the success rate of SDF was higher than interim restoration with GI filling (67% vs. 39%).	No details about the random allocation and attrition rate. Non-blinded study and duration of study was short.
	1 year					
				Active: lesion easily penetrated by probe		
			Gp 2: 30% SDF	Arrested: smooth, hard surfaces when probing	SDF was more effective than interim restoration with GI for arresting caries in primary teeth.	*High risk of bias*
						*Study quality (ADA): poor*
Chu et al., 2002 [13]	RCT	375 children	Gp 1: excavation plus 38% SDF once a year	Clinical examination criteria:	SDF groups (Gp 1, 2) had higher caries arrest rates than those of NaF groups (Gp 3, 4) and control. The respective mean numbers of arrested caries tooth surfaces in the five groups were 2.5, 2.8, 1.5, 1.5 and 1.3, respectively.	Ethical concern regarding the negative control group (no treatment)
	30 months	(aged 3–5 years)				
		Upper anterior primary teeth	Gp 2: 38% SDF once a year	Arrested caries: cavity with hard floor and walls		
						Only anterior primary teeth were involved. The generalizability of the results to posterior teeth was limited.
			Gp 3: excavation plus 5% NaF 4 times a year			
			Gp 4: 5% NaF 4 times a year			
			Gp 5: Control (no treatment)		SDF was effective in arresting dentin caries.	*Low risk of bias*
						*Study quality (ADA): good*
Lo et al., 1998 [9]	Longitudinal study	289 children aged 3–6 years	Gp 1: Regular oral health education session and daily tooth brushing with 1000 ppm fluoridated toothpaste	Clinical examination criteria:	Significant difference between the mean no. of arrested caries in Gp 1 and 2 which was 1.8 and 1.1, respectively.	Low attrition rate over 3 years. Potential confounders were evaluated. Although no random allocation was performed, comparable groups were assembled initially and maintained throughout the study.
	3 years	(168 children in intervention group, 121 children in the control group)		Arrested caries: dark brown to black in color with hard surface		
					At 3 years, 28% of the active dentin caries in Gp 1 children had become arrested while 12% of the caries were arrested in the control.	
			Gp 2: Control (no intervention)			
						*Moderate risk of bias*
						*Study quality (ADA): good*

All four included studies were conducted in a school setting in a non-fluoridated community, three were in China and one was in Brazil. Three randomized controlled trials investigated the effect of SDF application on dentin carious lesions compared to other interventions. One clinical trial compared the effect of daily brushing with 1000 ppm F dentifrice on caries arrest compared to a negative control (no intervention). Evaluation of clinical outcomes was based on clinical examination without taking radiographs. The therapeutic effect of these interventions was reported using the mean number and/or percentage of arrested caries tooth surfaces. Randomization was reported in most studies, often without much elaboration. Details of allocation concealment were not reported in any of the included studies. Blind assessment of outcome was impossible in most trials. No data were available about the intention to treat analysis for the RCT studies. Major adverse effects were neither systematically studied nor reported in the included SDF studies.

Among the four included studies, two randomized controlled trials compared the effectiveness of non-surgical treatment versus surgical treatment in arresting dentin caries in preschool children [14,15]. According to the ADA criteria, quality of one of these two trials was graded as “good” and the other as “poor”. The therapeutic interventions of both trials were similar, using either SDF or glass ionomer (GI) restorations under field settings. However, no caries removal was done prior to restoration placement (interim therapeutic restoration) in the study by dos Santos et al. [14], while the other trial removed soft caries using hand excavation and restored the cavity with flowable glass ionomer cements [15]. The 12-month results of these trials showed that SDF applied once a year exhibited a wide range of success rate, from 37% to 67%. Low success rates of restorations were found in both studies at the 12-month follow up, 29% [15] and 39% [14]. However, the 2-year results showed higher success rates of SDF applications and GI restorations [15]. Annual and semi-annual application of SDF solution could arrest active dentin caries with a success rate of 79% and 91%, respectively, while the annual paint-on of flowable GI could also arrest active dentin caries (82%) [15]. Arrest of active dentin caries could be enhanced by increasing the frequency of application from annually to every 6 months. Regarding the positive results of these two clinical trials, it should be noted that bias could occur since the materials used looked totally different.

The other two included clinical studies used topical fluoride agents to treat dentin caries, one with professionally applied fluoride (NaF and SDF) and another one tested the effectiveness of self-applied fluoride (toothpaste with 1000 ppm fluoride). Both studies were rated as ‘good’. The randomized controlled trial by Chu et al. [13] recruited only children with carious primary maxillary anterior teeth. This study examined the clinical effect of different topical fluoride regimens (5% NaF applied every 3 months and 38% SDF applied once a year) with and without excavation of carious tissues on caries arrest and prevention. The results indicated that SDF was substantially more effective than NaF and water (control) in arresting dentin caries. After 30 months, most active carious lesions treated with SDF had become arrested. No advantage of caries removal prior to topical fluoride application was found. Black staining, which is one of the disadvantages of using SDF to arrest caries, was reported. Despite this, the presence of black arrested caries lesions did not lead to an increase in parental dissatisfaction [13,15].

In the study by Lo et al. [9], a prevention program which included oral health education sessions and a daily toothbrushing exercise using fluoridated toothpaste (1000 ppm F) was provided to 168 children in a kindergarten. The control group was 121 children in two other kindergartens without a preventive program. Arrested caries were found in children from all three kindergartens, but significantly more so in the kindergarten with a daily toothbrushing exercise. At the third annual examination, 28% of the active dentin caries in children in the kindergarten with a daily toothbrushing exercise had become arrested while only 12% of the active caries in the control group children were arrested. The authors concluded that the use of simple prevention programs could stabilize the dental caries situation in young children living in communities where intensive use of trained dental personnel was not feasible. No adverse effects were reported.

Table 2 shows the the number of active caries surfaces that needed to be treated for one to benefit (become arrested) compared with control in the two included studies. Since there was no negative control group in the studies by dos Santos et al. [14] and Zhi et al. [15], no calculation of NNTs was made in these two studies. In the study by Chu et al. [13], The lower NNT (3.2) of the SDF groups reflects the higher effectiveness of SDF treatment compared to that of fluoride varnish (NNT being 10–25). In the study by Lo et al. [9], the NNT was 6.3 which was calculated from the beneficial effect of toothbrushing program with fluoridated toothpaste compared with control.

**Table 2:** **Proportion benefiting from the intervention and number needed to treat in the two included studies**

**Study**	**Intervention (test group)**	**Caries arrest rate in test group**	**Caries arrest rate in control group**	**Proportion benefiting from intervention**	**Number needed to treat**
		**P (A)**	**P (B)**	**P(C) = P(A) - P(B)**	**NNT = 1/P(C)**
Chu et al. 2002 [13]	38% SDF + excavation	0.65	0.34	0.31	3.2
	38% SDF	0.65	0.34	0.31	3.2
	5% NaF + excavation	0.38	0.34	0.04	25
	5% NaF	0.44	0.34	0.10	10
Lo et al. 1998 [9]	Brushing with fluoridated toothpaste	0.28	0.12	0.16	6.3

## Discussion

Over the years, efforts have been made to improve the quality of clinical studies such as adopting the SPIRIT 2013 statement [31], CONSORT 2010 statement [32] or to strengthen the reporting of observational studies with STROBE guidelines [33]. It was expected that much information could be found to provide evidence for the clinical efficacy of non-surgical treatment of dentin caries in young children. However, few studies were included in this review, despite the inclusion criteria were set as broad as possible. The lack of quality clinical trials in primary teeth was also found in other systematic reviews [2,34]. In addition, some included studies were assessed as at moderate or high risk of bias. Blinding of outcome assessment was not always possible, especially when comparing the results of restorative and topical fluoride treatments. There was no reporting on the two criteria “intention to treat analysis” and “allocation concealment”. The findings would have been more convincing if the trials had been designed, analyzed, and interpreted following the standard protocol items for clinical trials.

Since two of the co-authors of this review (CHC and ECML) had conducted a number of clinical studies in this field, in order to minimize selection bias, two independent reviewers (DD and MJ) who did not involve in the clinical studies conducted the literature search and they adhered strictly to the search criteria in the review process. It should be noted that papers by the co-authors of this review were finally included and there may be a bias. Ideally, a systematic review without language restriction should be conducted. However, due to limitation of resources in this study, only papers published in English were reviewed and this may lead to a reporting bias because some early clinical studies on the use of SDF were conducted in Japan and in China [35].

Our results showed congruence with previous reviews supporting the beneficial effect of SDF solution. A systematic review by Rosenblatt et al. [16] evaluating two clinical studies concluded that SDF is more effective than fluoride varnish and may be valuable caries-preventive intervention. A more recent systematic review by Chen et al. [36] confirmed the efficacy of SDF for treatment and prevention of dental caries in children aged 0–18 years. The benefit of using SDF was not confined to child populations only. A recent review summarized the effectiveness of root caries preventive agents and made recommendations for annual application of 38% SDF in adults and vulnerable elderly [37].

The inclusion of three SDF studies in the present review considerably strengthened the evidence of the effectiveness of SDF in arresting dentin caries in preschool children. Three studies reported significantly higher success rates of SDF treatment (65-91%) compared with no treatment (34%), sodium fluoride varnish (38-44%) and interim GI restorations (39-82%). It was found that application of SDF had a superior effect on caries arrest than interim GI restorations. Nevertheless, it should be noted that the restorative treatment was done under the limitations of a field setting. In addition, no caries removal was done prior to restoration placement in the clinical trial by dos Santos et al., probably leading to lower retention rates [14]. Another study used flowable GI restorations which generally have lower strength compared to other restorative materials such as composite resin or amalgam [15]. Therefore, these results should be interpreted with caution, as the findings may not reflect the success rates of restorations done under a standard clinical setting.

Strong evidence from numerous narrative and systematic reviews supports the effectiveness of fluoride varnish in preventing dental caries. Regarding the therapeutic effect of fluoride varnish, a systematic review concluded that fluoride varnish was one of the fluoride interventions which seemed to have the most consistent effect in retarding the progression of noncavitated carious lesions [38]. Despite this, the results of this review indicate that application of NaF varnish (4 times a year) seems to be less effective than application of SDF solution (once a year) in arresting active dentin caries in primary teeth in preschool children.

Although SDF has been in use for a few decades without serious adverse effect, the safety issue of applying high concentration fluoride is still equivocal, especially in young children. The possibility of chronic and acute toxicity through the use of SDF has been debated [39,40]. Since the maximum follow-up period of the included studies in this review was less than 3 years, there was no report on the risk of fluorosis in children receiving SDF treatment. Regarding the potential risk associated with silver ingestion, preliminary data in adults show that occasional use of SDF is well below the concentrations associated with toxicity [41]. However, without data in young children, this possibility cannot be excluded and one needs to pay attention to the safety aspect when applying high concentration fluoride and silver agents to young children. To identify the effectiveness and limitations associated with SDF use, a longer observation period is required, and adverse effects should be systematically studied and reported.

The current review found limited evidence to support the use of SDF as an effective therapeutic agent for treating dentin caries in young children. Additional advantages include being a simple, non-invasive and cost-effective treatment compared to restorations and other fluoride agents such as NaF varnish. However, dark staining of the caries lesions after SDF application is common. Studies on how to overcome blackening of lesions caused by SDF treatment are needed.

There is limited evidence showing the benefits of daily toothbrushing with 1000 ppm fluoride toothpaste in arresting or slowing down the progression of active dentin caries in young children. These findings are in agreement with those of Kidd and Fejerskov [42] which suggested control of oral biofilm is a treatment for dental caries, the most important measure being to disturb the biofilm mechanically using a fluoride-containing toothpaste. Study findings have confirmed the preventive benefits of using toothpaste with fluoride concentrations of 1000 ppm and above, when compared with placebo, in children and adolescents [43,44]. Although fluoride toothpaste has a pronounced effect in preventing dental caries, evidence for effectiveness on arresting dental caries especially dentin caries is scarce. At the full text stage, this study found one study reporting a superior anticaries effect of 1100 ppm F dentifrices on enamel caries progression when compared to the 500 ppm F dentifrice [45]. Nevertheless, there were insufficient data to determine the effectiveness of different fluoride concentrations in fluoride toothpastes on treating dentin caries in young children.

A recent systematic review concluded that sealants and resin infiltrates have a potential benefit in halting or arresting dental caries [38]. One of the excluded studies in this review reported a similar finding in preschool children that sealing enamel/dentin proximal caries with bonding agents halted dentin caries progression [46]. However, this study was finally excluded due to the small sample size of dentin carious lesions. So, there is still insufficient evidence showing that there is a difference in caries stabilization between sealing proximal dentin caries and providing instructions on flossing to preschool children.

Systematic searches of the literature found very few clinical studies on the efficacy of xylitol, chlorhexidine, CPP-APC and ozone therapy on caries arrest in primary teeth in young children and the results were inconclusive. Xylitol chewing gum has been found to be effective in arresting dentin caries in primary teeth, but the age of participants was initially 6 years or older [47]. Thus, the study was excluded at the final stage. Although the habitual use of xylitol or polyol chewing gum is an effective adjunct in caries prevention for children aged 5 years or older with high caries risk [48], choking hazard is a major concern for young children. Findings of this review are consistent with those of Rethman et al. [49] which show that there is insufficient evidence to support recommendations for the use of xylitol chewing gum, candy or lozenges by children younger than 5 years.

CPP-ACP has been incorporated into products such as commercial mouthwashes, sugar-free chewing gums or dental creams [50]. A systematic review with meta-analysis concluded that there was clinical evidence for enamel remineralization and caries prevention by regular use of products containing CPP-ACP [51]. Conversely, a randomized clinical trial conducted on preschool children found that daily application of 10% w/v CPP-ACP paste on school days had no significant effect in preventing caries in the primary dentition [52]. So far, no clinical studies were found regarding the use of CPP-ACP in arresting dentin caries in preschool children. Chlorhexidine (CHX) has been studied for its potential to prevent and control dental caries. Several systematic reviews with conflicting results on the effectiveness of CHX varnish have been published [53,54]. Most trials comparing the preventive effect of CHX varnish were conducted on school children, adolescent or adults. The present systematic review did not find any evidence regarding the efficacy of CHX varnish on dentin caries progression in preschool children. Regarding the application of ozone gas, the results of the present review are consistent with those of previous reviews [55,56], i.e. there is no clinical evidence for the use of ozone in arresting dentin caries in primary teeth in preschool children.

Regarding the outcome assessment of all included studies, the criteria used to classify active or arrested caries lesions are based on visual inspection and tactile sensation using a probe. It should be noted that lesion activity assessment at one time point only tells about the probability or risk of progression [57]. Although the inter- and intra-reliability of all included studies in this review were high (Kappa value over 0.85), a major concern is a lack of an accepted clinical gold standard which can reliably differentiate between active and arrested lesions. Research to develop an accurate, objective and reproducible lesion activity measurement is needed.

## Conclusion

There is limited evidence to support the effectiveness of SDF applications once/twice a year and that of daily toothbrushing with fluoride toothpaste in arresting or slowing down the progression of active dentin caries in primary teeth in preschool children. Due to the small numbers of included studies, more well-designed randomized controlled trials on young children are required to confirm or to refute these findings.

## Abbreviations

ECC: Early childhood caries
SDF: Silver diammine fluoride
NaF: Sodium fluoride
CPP-ACP: Casein phosphopeptide - amorphous calcium phosphate
CHX: Chlorhexidine
MeSH: Medical subject headings
ADA: The American Dental Association
RCT: Randomized control trial
GI: Glass ionomers
SPIRIT: Standard Protocol Items, Recommendations for Interventional Trials
Consort: CONsolidated Standards of Reporting Trials
STROBE: STrengthening the Reporting of OBservational studies in Epidemiology

## References

[1] Finucane D (2012). Rationale for restoration of carious primary teeth: A review. Eur Arch Paediatr Dent.

[2] Yengopal V, Harneker SY, Patel N, Siegfried N. Dental fillings for the treatment of caries in the primary dentition. Cochrane Database Syst Rev. 2009; Issue 2. Art. No.: CD004483. doi:10.1002/14651858.CD004483.pub2. 10.1002/14651858.CD004483.pub2 19370602

[3] Chu CH, Ho PL, Lo EC (2012). Oral health status and behaviours of preschool children in Hong Kong. BMC public health.

[4] Frencken JE, Peters MC, Manton DJ, Leal SC, Gordan VV, Eden E (2012). Minimal intervention dentistry for managing dental caries - a review: report of a FDI task group. Int Dent J.

[5] Pine CM, Adair PM, Burnside G, Nicoll AD, Gillett A, Borges-Yanez SA (2004). Barriers to the treatment of childhood caries perceived by dentists working in different countries. Community Dent Health.

[6] Tickle M, Milsom K, King D, Kearney-Mitchell P, Blinkhorn A (2002). The fate of the carious primary teeth of children who regularly attend the general dental service. Br Dent J.

[7] Foley J (2006). Alternative treatment strategies for carious primary teeth: an overview of the evidence. Eur Arch Paediatr Dent.

[8] Evans RW, Dennison PJ (2009). The Caries Management System: an evidence-based preventive strategy for dental practitioners. Application for children and adolescents. Aus Dent J.

[9] Lo EC, Schwarz E, Wong MC (1998). Arresting dentine caries in Chinese preschool children. Int J Paediatr Dent.

[10] Baysan A, Lynch E, Ellwood R, Davies R, Petersson L, Borsboom P (2001). Reversal of primary root caries using dentifrices containing 5,000 and 1,100 ppm fluoride. Caries Res.

[11] Llodra JC, Rodriguez A, Ferrer B, Menardia V, Ramos T, Morato M (2005). Efficacy of silver diamine fluoride for caries reduction in primary teeth and first permanent molars of schoolchildren: 36-month clinical trial. J Dent Res.

[12] Liu BY, Lo ECM, Chu CH, Lin HC (2012). Randomized Trial on Fluorides and Sealants for Fissure Caries Prevention. J Dent Res.

[13] Chu CH, Lo EC, Lin HC (2002). Effectiveness of silver diamine fluoride and sodium fluoride varnish in arresting dentin caries in Chinese pre-school children. J Dent Res.

[14] dos Santos Jr VE, De Vasconcelos FMN, Ribeiro AG, Rosenblatt A (2012). Paradigm shift in the effective treatment of caries in schoolchildren at risk. Int Dent J.

[15] Zhi QH, Lo EC, Lin HC (2012). Randomized clinical trial on effectiveness of silver diamine fluoride and glass ionomer in arresting dentine caries in preschool children. J Dent.

[16] Rosenblatt A, Stamford TC, Niederman R (2009). Silver diamine fluoride: a caries “silver-fluoride bullet”. J Dent Res.

[17] Mertz-Fairhurst EJ, Schuster GS, Williams JE, Fairhurst CW (1979). Clinical progress of sealed and unsealed caries. Part II: Standardized radiographs and clinical observations. J Prosth Dent.

[18] American Academy of Pediatric Dentistry (2012). Guideline on pediatric restorative dentistry. Pediatr Dent.

[19] Meyer-Lueckel H, Bitter K, Paris S (2012). Randomized controlled clinical trial on proximal caries infiltration: three-year follow-up. Caries Res.

[20] Li J, Xie X, Wang Y, Yin W, Antoun JS, Farella M (2014). Long-term remineralizing effect of casein phosphopeptide-amorphous calcium phosphate (CPP-ACP) on early caries lesions in vivo: a systematic review. J Dent.

[21] Fontana M, Gonzalez-Cabezas C (2012). Are we ready for definitive clinical guidelines on xylitol/polyol use?. Adv Dent Res.

[22] Mäkinen KK, Hujoel PP, Bennett CA, Isotupa KP, Mäkinen PL, Allen P (1996). Polyol chewing gums and caries rates in primary dentition: a 24-month cohort study. Caries Res.

[23] Milgrom P, Ly KA, Tut OK, Mancl L, Roberts MC, Briand K (2009). Xylitol pediatric topical oral syrup to prevent dental caries: a double-blind randomized clinical trial of efficacy. Arch Pediatr Adol Med.

[24] Edelstein BL (2009). Solving the problem of early childhood caries: a challenge for us all. Arch Pediat Adol Med.

[25] Hobdell M, Petersen PE, Clarkson J, Johnson N (2003). Global goals for oral health 2020. Int Dent J.

[26] Higgins JP, Altman DG, Gotzsche PC, Juni P, Moher D, Oxman AD (2011). The Cochrane Collaboration’s tool for assessing risk of bias in randomised trials. BMJ.

[27] Center for Evidence-Based Dentistry, American Dental Association. ADA Clinical Recommendations Handbook; 2011. http://ebd.ada.org/en/evidence/guidelines (Accessed February 27, 2014)

[28] Laupacis A, Sackett DL, Roberts RS (1988). An assessment of clinically useful measures of the consequences of treatment. N Engl J Med.

[29] Lo EC, Chu CH, Lin HC (2001). A community-based caries control program for pre-school children using topical fluorides: 18-month results. J Dent Res.

[30] Wong MC, Lam KF, Lo EC (2005). Bayesian analysis of clustered interval-censored data. J Dent Res.

[31] Chan AW, Tetzlaff JM, Altman DG, Laupacis A, Gotzsche PC, Krleza-Jeric K (2013). SPIRIT 2013 statement: defining standard protocol items for clinical trials. Ann Intern Med.

[32] Schulz KF, Altman DG, Moher D (2010). CONSORT 2010 statement: updated guidelines for reporting parallel group randomised trials. PLoS medicine.

[33] von Elm E, Altman DG, Egger M, Pocock SJ, Gotzsche PC, Vandenbroucke JP (2008). The Strengthening the Reporting of Observational Studies in Epidemiology (STROBE) statement: guidelines for reporting observational studies. J Clin Epidemiol.

[34] Ricketts D, Lamont T, Innes NP, Kidd E, Clarkson JE (2013). Operative caries management in adults and children. Cochrane Database Syst Rev.

[35] Chu CH, Lo ECM (2008). Promoting caries arrest in children with silver diamine fluoride. Oral Health Prev Dent.

[36] Chen A, Cho M, Kichler S, Lam J, Liaque A, Sultan S (2012). Silver diamine fluoride: an alternative to topical fluorides. J Can Dent Assoc.

[37] Gluzman R, Katz RV, Frey BJ, McGowan R (2013). Prevention of root caries: a literature review of primary and secondary preventive agents. Spec Care Dentist.

[38] Tellez M, Gomez J, Kaur S, Pretty IA, Ellwood R, Ismail AI (2012). Non-surgical management methods of noncavitated carious lesions. Community Dent Oral Epidemiol.

[39] Gotjamanos T (1997). Safety issues related to the use of silver fluoride in paediatric dentistry. Aust Dent J.

[40] Neesham DC (1997). Fluoride concentration in AgF and dental fluorosis. Aust Dent J.

[41] Vasquez E, Zegarra G, Chirinos E, Castillo JL, Taves DR, Watson GE (2012). Short term serum pharmacokinetics of diammine silver fluoride after oral application. BMC Oral Health.

[42] Kidd E, Fejerskov O (2013). Changing concepts in cariology: forty years on. Dent Update.

[43] Twetman S (2009). Caries prevention with fluoride toothpaste in children: an update. Eur Arch Paediatr Dent.

[44] Walsh T, Worthington HV, Glenny AM, Appelbe P, Marinho VC, Shi X. Fluoride toothpastes of different concentrations for preventing dental caries in children and adolescents. Cochrane Database Syst Rev. 2010; Issue 1. Art. No.: CD007868. doi:10.1002/14651858.CD007868.pub2 10.1002/14651858.CD007868.pub2 20091655

[45] Lima TJ, Ribeiro CC, Tenuta LM, Cury JA (2008). Low-fluoride dentifrice and caries lesion control in children with different caries experience: a randomized clinical trial. Caries Res.

[46] Martignon S, Tellez M, Santamaria RM, Gomez J, Ekstrand KR (2010). Sealing distal proximal caries lesions in first primary molars: efficacy after 2.5 years. Caries Res.

[47] Makinen KK, Makinen PL, Pape HR, Allen P, Bennett CA, Isokangas PJ (1995). Stabilisation of rampant caries: polyol gums and arrest of dentine caries in two long-term cohort studies in young subjects. Int Dent J.

[48] Ly KA, Milgrom P, Rothen M (2008). The potential of dental-protective chewing gum in oral health interventions. J Am Dent Assoc.

[49] Rethman MP, Beltran-Aguilar ED, Billings RJ, Hujoel PP, Katz BP, Milgrom P (2011). Nonfluoride caries-preventive agents: executive summary of evidence-based clinical recommendations. J Am Dent Assoc.

[50] Cochrane NJ, Cai F, Huq NL, Burrow MF, Reynolds EC (2010). New approaches to enhanced remineralization of tooth enamel. J Dent Res.

[51] Yengopal V, Mickenautsch S (2009). Caries preventive effect of casein phosphopeptide-amorphous calcium phosphate (CPP-ACP): a meta-analysis. Acta Odontol Scand.

[52] Sitthisettapong T, Phantumvanit P, Huebner C, Derouen T (2012). Effect of CPP-ACP paste on dental caries in primary teeth: a randomized trial. J Dent Res.

[53] Twetman S (2004). Antimicrobials in future caries control? A review with special reference to chlorhexidine treatment. Caries Res.

[54] James P, Parnell C, Whelton H (2010). The caries-preventive effect of chlorhexidine varnish in children and adolescents: a systematic review. Caries Res.

[55] Rickard GD, Richardson R, Johnson T, McColl D, Hooper L. Ozone therapy for the treatment of dental caries. Cochrane Database Syst Rev. 2004; Issue 3. Art. No.: CD004153. doi:10.1002/14651858.CD004153.pub2. 10.1002/14651858.CD004153.pub2 15266519

[56] Burke FJ (2012). Ozone and caries: a review of the literature. Dent Update.

[57] Ekstrand KR, Zero DT, Martignon S, Pitts NB (2009). Lesion activity assessment. Monogr Oral Sci.

